# Phylogenetic and expression analyses of HSF gene families in wheat (*Triticum aestivum* L.) and characterization of *TaHSFB4-2B* under abiotic stress

**DOI:** 10.3389/fpls.2022.1047400

**Published:** 2023-01-25

**Authors:** Liu Yun, Yan Zhang, Shi Li, Jingyu Yang, Changyu Wang, Lanjie Zheng, Li Ji, Jiaheng Yang, Linhu Song, Yong Shi, Xu Zheng, Zhiyong Zhang, Jie Gao

**Affiliations:** ^1^ State Key Laboratory of Wheat and Maize Crop Science, and Center for Crop Genome Engineering, College of Agronomy, Henan Agricultural University, Zhengzhou, Henan, China; ^2^ College of Life sciences, Neijiang Normal University, Neijiang, Sichuan, China

**Keywords:** *HSF*, *TaHSFB4-2B*, wheat, tissue specific expression, overexpression, abiotic stress

## Abstract

The heat shock transcription factors (HSFs) family is widely present in eukaryotes including plants. Recent studies have indicated that HSF is a multifunctional group of genes involved in plant growth and development, as well as response to abiotic stresses. Here we combined the bioinformatic, molecular biology way to dissect the function of Hsf, specifically HsfB4 in wheat under abiotic stresses. In this study, we identified 78 TaHSF genes in wheat (Triticum aestivum) and analyzed their phylogenetic relationship and expression regulation motifs. Next, the expression profiles of TaHSFs and AtHSFs were analyzed in different tissues as well as in response to abiotic stress. Furthermore, to explore the role of HSFB4 in abiotic stress response, we cloned TaHSFB4-2B from the wheat variety, Chinese Spring. Subcellular localization analysis showed that TaHSFB4-2B was localized in the nucleus. In addition, We observed TaHSFB4-2B was highly expressed in the root and stem, its transcription was induced under long-term heat shock, cold, and salinity stress. Additionally, overexpression of TaHSFB4-2B suppressed seed germination and growth in Arabidopsis with salinity and mannitol treatment. It also modulated the expression of stress-responsive genes, including AtHSP17.8, AtHSP17.6A, AtHSP17.6C, CAT2, and SOS1, under both normal and stress conditions. From these finding, we propose that TaHSFB4-2B act as a negative regulator of abiotic stress response in the plant.

## Introduction

The abiotic stresses result in growth retardation, quality reduction, and yield loss of crop plants (Haider et al., 2022). For example, high temperature is found to significantly reduce crop yields (Schlenker and Roberts, 2009). Low temperature affects plant metabolism by directly inhibiting metabolic enzymes. Cold acclimation causes an increase in 75% of the 434 total metabolites detected in arabidopsis (Cook et al., 2004; Kaplan et al., 2004). Salinity leads to ionic toxicity, hypertonic stress, and oxidative damage (Zhu, 2002), while drought alters the growth and structure of plant roots, resulting in early flowering or growth retardation, and reduces yield (Gupta et al., 2020).

HSFs are the key regulators of heat stress response in plants. They specifically bind to highly conserved heat shock elements (*HSE*) to form transcriptional complexes that regulate the expression of downstream heat shock proteins (HSPs) (Lin et al., 2011). Based on the variations in the highly conserved functional domain, plant HSFs are categorized into three classes: HSFA, HSFB, and HSFC (Nover et al., 2001; Baniwal et al., 2004). HSFAs contain an AHA motif in the C-terminal activating peptide that participates in transcriptional activation (Czarnecka-Verner et al., 2004). Unlike HSFAs, HSFBs and HSFCs do not have an activation domain. As a result, they were presumed to be devoid of transcriptional activity (von Koskull-Doring et al., 2007).

In recent years, deducing the functions of HSFBs has become a research hotspot. In Arabidopsis, the *HSFB* subfamily is comprised of five members: *AtHSFB1*, *AtHSFB2a*, *AtHSFB2b*, *AtHSFB3*, and *AtHSFB4*. *AtHSFB1* and *AtHSFB2b* are transcriptional inhibitors of heat-induced *HSFs*, which are involved in the positive regulation of acquired heat tolerance in the plant (Ikeda et al., 2011). AtHSFB4 primarily works in root stem cells and controls the development of cells in the surrounding layers. Arabidopsis *scz*, a mutant of *AtHsfB4*, showed an abnormal division of root peripheral cells, significantly increased root hair and shortened root length compared to wild-type (WT) plants (Mylona et al., 2002; Mylona et al., 2002; ten Hove et al., 2010; Begum et al., 2013). In rice, transgenic lines overexpressing OsHSFB4d showed enhanced disease resistance to bacterial leaf streak (BLS) and bacterial blight (BB) (Yang et al., 2020). In addition, heat, cold, and oxidative stress induced the transcription of *OsHSFB4a*, *OsHSFB4b*, and *OsHSFB4d* in rice (Mittal et al., 2009), indicating that the *OsHSFB4* subfamily of genes is likely to be involved in heat and cold, as well as other stress responses.

In the current study, we analyzed the phylogenetic relationship among TaHSF proteins and the collinearity of *TaHSF* genes. The tissue-specific expression of *TaHSFs* and their responses to heat, cold, salinity, mannitol-induced drought stress, and exogenous ABA were investigated. Given the wide existence and diverse functions of *HSFB4* genes, we cloned the closest ortholog of *AtHSFB4* in wheat through homology-based cloning (http://plants.ensembl.org/index.html). The gene was designated *TaHSFB4-2B* based on its location on chromosome 2 of wheat subgenome B. We next analyzed the structural characteristics, subcellular localization, tissue-specific expression pattern, and expression profiles of *TaHSFB4-2B* under various abiotic stresses. Further, the transgenic lines overexpressing *TaHSFB4-2B* in arabidopsis were generated and their response to NaCl and mannitol-induced drought stress was evaluated. Taken together the findings, we concluded that *TaHSFB4-2B* acts as a negative regulator of heat and drought stress response in arabidopsis.

## Materials and methods

### Sequence and bioinformatics analysis

The amino acid sequences of *HSF* genes in arabidopsis (*Arabidopsis thaliana*), wheat (*Triticum aestivum*), soybean (*Glycine max*), tomato (*Solanum lycopersicum*), potato (*Solanum tuberosum*), rape (*Brassica napus*), rice (*Oryza sativa*), and corn (*Zea mayz*) were downloaded from Ensembl plants (http://plants.ensembl.org/index.html). MEGA-X (version 10.1.8) software was used for multiple sequence alignment and phylogenetic analysis was conducted using neighbor-joining method (Bootstrap test method was adopted and the replicate was set to 1000) (**Tables S1**, **2**). Based on the information of wheat genome database, wheat *HSF* genes were mapped to different chromosomes, and the gene duplication events of *HSF* genes in wheat were visualized by TBtools (https://github.com/CJ-Chen/TBtools/releases). *TaHSFB4-2B* sequence was downloaded from Ensembl plants database. The TaHSFB4-2B protein domain was examined using the SMART online tool (http://smart.embl-heidelberg.de/). The *TaHSFB4-2B* gene structure was created using the GSDS website (http://gsds.cbi.pku.edu.cn/).

### Analysis of induced abiotic stress *cis*-regulting elements of *AtHSFs* and *TaHSFs*

To further identify the putative induced abiotic stress *cis*-regulatory elements of the promoter regions of the *AtHSFs* and *TaHSFs* genes, 2-kb upstream sequences of *AtHSFs* and *TaHSFs* genes were obtained by using TBtools (https://github.com/CJ-Chen/TBtools/releases). The various putative *cis*-regulatory elements of these sequences were further analyzed using PlantCARE databases (http://bioinformatics.psb.ugent.be/webtools/plantcare/html/).

### Expression analysis of the *AtHSFs* and *TaHSFs* gene family from RNA-Seq data

To further analyze the spatiotemporal expression patterns of *AtHSF* and *TaHSF*, the transcriptomic data were downloaded from the Wheat eFP Browser (http://bar.utoronto.ca/efp/cgi-bin/efpWeb.cgi) and ExpVIP (http://www.wheat-expression.com/), respectively. According to the expression databases of arabidopsis and wheat, we analyzed *AtHSF* and *TaHSF* expression patterns in different tissues, different developmental stages and under different abiotic stresses. The expression levels of *AtHSF* and *TaHSF* genes were then drawn into heatmaps by TBtools (https://github.com/CJ-Chen/TBtools/releases).

### Gene cloning and construction of transgenic plants

Total RNAs were isolated from young roots of 14-day-seedling wheat (Chinese Spring) and reversely transcribed into cDNA. Specific primers of *TaHSFB4-2B* were designed for its coding sequence amplication. Briefly, a 50 μL PCR reaction contained approximately 200 ng of cDNA, 25 μL of 2×PrimeSTAR HS (Premix), and 100 nM primers. The PCR programs were conducted following manufacturer’s instruction with an annealing temperature of 58°C for 30 seconds. The PCR products were purified from agarose gel, and then *35S*::*TaHSFB4-2B-GFP* vector was transformed into *Agrobacterium tumefaciens* GV3101 strain, which was then used for transformation of arabidopsis (Col-0) by floral dip method. T_0_ transgenic lines were screened by 1/2 MS (Murashige and Skoog) medium with 50 mg/L Kan (Kanamycin) and green fluorescent protein signal was observed by fluorescence microscope. Homozygous T_3_ plants were obtained by successive self-crossing after screening for further research.

### Protoplast isolation and transformation in wheat and arabidopsis protoplast

Wheat was cultured in the greenhouse at 25 ± 2°C with a light of 14-16 h/d for 2 weeks. Young leaves were detached from plants by a scissor and carefully sliced into 0.5-1 mm strips by sharp surgical blade. Then the sample strips were gently submerged into the 0.6 M mannitol for 10 min. After filtered, the samples were transferred into petri dish containing 50 ml enzyme buffer (1.5% cellulase R10, 0.75% macerozyme R10, 0.6 M mannitol, 10 mM MES, 10 mM CaCl_2_, 0.1% BSA, pH=5.7). Then the samples were penetrated under vacuum 15 Kpa for 30 min. The petri dish was fixed on a shaker at a speed of 10-20 RPM for 5 h. 30 ml W5 (150 mM NaCl, 125 mM CaCl_2_, 5 mM KCl, 2 mM MES, pH=5.7) were used for dilution of the protoplast and the solution were filtered by 75 um nylon membrane. Protoplast was collected by centrifuging at a speed of 100 rcf (g) for 3min. The protoplast was resuspended by 10 ml W5 and incubated on ice for 30 min. Supernatant was removed and MMG (0.4 M mannitol, 15 mM MgCl_2_, 4 mM MES) was used to dilute protoplast to a concentration of 2x10^5^/ml-1x10^6^/ml. For transfection 100ul protoplasts were transfected with 10-20ug plasmid and incubated in dark at 25°C for 12-16h and used for further analysis.

Arabidopsis were cultured in chamber with the condition of 22°C and 16h/8h light/dark cycle until 4 weeks. Then fresh leaves were processed as stated above as wheat leaves. The experiment process and solution preparation referred to Barnes et al. (2019).

### TaHSFB4-2B protein subcellular location

Either *35S*::*TaHSFB4-2B-GFP* or *35S*::*GFP* was co-transformed with nuclear marker *35S*::mCherry-IMP4 into wheat protoplast; *35S*::*TaHSFB4-2B-GFP* was co- transformed with either free *35S*::mCherry or nuclear marker *35S*::mCherry-IMP4 into arabidopsis protoplast. Then, a confocal microscope was used for imaging.

In addition, *35S*::*TaHSFB4-2B-eGFP* and nuclear localization marker *35S*::*mCherry-IMP4* was co-transformed into 4-week-old *Nicotiana benthamiana* leaves mediated by *Agrobacterium tumefaciens* EHA105 strain. The *Agrobacterium tumefaciens* with plasmid *vectors* was cultured in 3 mL liquid LB medium with Kan and Rifampicin (Rif) antibiotics at 28°C and 250 RPM rotation for about 16 h until OD_600_ = 1-2, and then 5 uL of the culture was inoculated to 10 mL of fresh liquid LB medium (50 mg/L Kan, 50 mg/L Rif, 10 mm MES, 20 μM AS), which was followed by incubation at 28°C and 250 RPM for about 16 h until OD_600_ = 1. The culture medium was centrifugated at 4 000 RPM for 10 min. After the supernatant was removed, the pellets were resuspended in solution with solution containing 10 mM MES, 150 μM AS, and 10mM MgCl_2_, and adjusted OD_600_ to 1. Tobacco leaves were injected with the bacterial solution, and then set aside in the dark room with room temperature for 3 h. Then the tobacco plants were cultured in the dark for 1 d and then grown normally for another 1 d. Confocal microscopy was performed with laser-scanning confocal imaging system.

### Wheat materials and stress treatment

Wheat variety Chinese Spring was used in this study. Wheat seeds were selected with same size and full particles, then were disinfected them with 20% hypochlorous acid for 20 min, and rinsed with sterile water 5 times. The selected seeds were placed with the ventral groove downward in the petri dish, and covered by wet filter paper, and cultured in the incubator for 3 d in the dark. After germination, the seeds were wrapped in sponge and then cultured in the whole wheat culture medium [0.1 mM Ca(NO_3_)_2_, 0.2 mM KH_2_PO_4_, 1 mM MgSO_4_·7H_2_O, 1.5 mM KCl, 1.5 mM CaCl_2_, 1 μM H_2_BO_3_, 5 μM (NH_4_)6Mo_7_O_2_·H_2_O, 0.5 μM CuSO_4_·5H_2_O, 1 μM ZnSO_4_·7H_2_O, 1 μM MnSO_4_·H_2_O, 100 μM Fe(III)-EDTA, pH7.0] with a plant distance of 10 cm at 25°C, 12 h light/12 h dark for 14 days. The medium was changed once every three days. After that, the seedings were treated with high temperature (37°C) and low temperature (4°C). For mannitol-induced dehydration simulating drought or salinity stress, the seedlings were transferred into the whole wheat culture medium with 300 mM mannitol or 200 mM NaCl. Wheat leaves and roots were sampled at the time point of 0 h, 1 h, 2 h and 6 h after treatment and stored respectively at -80°C.

### Stress tolerance analysis of transgenic arabidopsis

The homozygous seeds of T_3_ transgenic line were used for phenotypic analysis. Arabidopsis seeds were sterilized with 30% hypochlorous acid and 2‰ TritonX-100 for 10 min, and rinsed with sterile water for 5 times, then germinated in the dark at 4°C for 3 d. The germinated arabidopsis seeds were grown on 1/2 MS with additional 100 mM NaCl, 150 mM NaCl, 250 mM mannitol or 300 mM mannitol respectively, and were cultured at 22°C for 9 d (16 h light/8 h dark). Wild-type or over-expression seedlings treated by salinity or mannitol was phenotyped, and samples were harvested under stresses, and then frozen in liquid N2 for future processing.

### Expression analysis by RT-qPCR

The total RNA was extracted by Trizol (Tiangen, Beijing) following the manufacturer’s instructions with minor modification. The extracted RNA was reversely transcribed into cDNA according to the instructions of reverse transcription Kit (Takara, Dalian). The cDNA was diluted to a concentration of 400 ng/μL. Gene specific primers were designed for quantitative real-time PCR analysis (LC480, Roche, USA) and Actin was selected as the internal reference (**Table S3**). The PCR program was performed in three biological replicates and three technical replicates for each sample. Briefly, a 20-μL PCR reaction contained approximately 100 ng of cDNA, 10 μL of SYBR solution, and 200 nM primers. The 2^−ΔΔCt^ method was used for statistical analysis.

### Statistical analysis

In this study, the statistical analysis was reported as means ± SD with significance determined by Student’s t-test or ANOVA at least three replicates. Significance levels are marked as: *P<0.05, **P<0.01, non-significant (n.s.), *P*>0.05. Least Significant Difference (LSD) was used to compare *TaHSFB4-2B* tissue specific expression in wheat, letters (‘a-d’) indicate the statistical differences between different tissues determined by LSD (*P*<0.05) of variance (ANOVA) method. Same letters: no significant difference, and different letters: significant difference between the two groups.

## Results

### Phylogenetic and collinearity analyses of TaHSF family members

**Figure 1:**
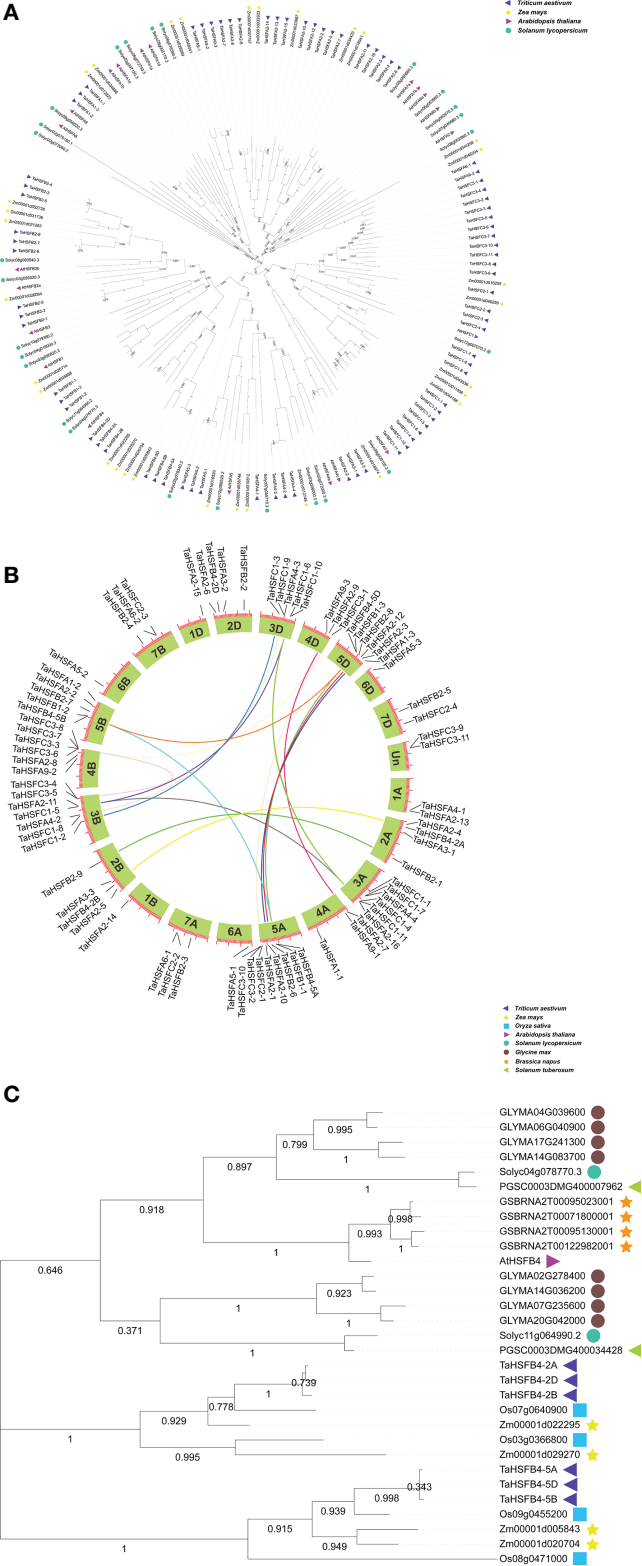
Neighbor-joining phylogenetic tree and synteny analysis of TaHSF families. **(A)** Neighbor-joining phylogenetic tree of *Triticum aestivum* (*Ta*), *Arabidopsis thaliana* (*At*), *Zea mays* (*Zm*) and *Solanum lycopersicum* (*Sl*) HSF families. TaHSFB4-2A, TaHSFB4-2B, and TaHSFB4-2D were marked in red. **(B)** Synteny analysis of *TaHSF* genes. **(C)** Phylogenetic analysis and amino acid sequence alignment of HSFB4s in *Ta*, *At*, *Zm*, *Sl*, *Oryza sativa* (*Os*), *Glycine max* (*Gm*), *Solanum tuberosum* (*St*) and *Brassica napus* (*Bn*).

We searched the Ensembl database (http://plants.ensembl.org/index.html) and found that there are 78 *HSF* genes in wheat, including 34 *TaHSFAs*, 18 *TaHSFBs*, and 26 *TaHSFCs*. To investigate their evolutionary relationships, the amino acid sequences of 78 TaHsfs were obtained, along with 31, 21, and 26 protein sequences of HSFs from widely cultivated monocotyledon maize (*Zea mays*) and dicotyledons Arabidopsis (*Arabidopsis thaliana*) and tomato (*Solanum lycopersicum*), respectively. Based on the phylogenetic analysis, HSF proteins from all four plants were classified into three subfamilies, *viz.* - HSFA-C, and the phylogenetic relationship between corresponding homologous proteins from each subfamily was more significant within monocotyledon crops or dicotyledon than between them (**Figure 1A**).

Gene collinearity analysis is an important approach to understand the evolutionary history of a genome (Lynch and Conery, 2000; Wang et al., 2012). To elucidate the evolutionary history of wheat *HSF* genes, the collinearity map of *TaHSFs* was constructed using TBtools (https://github.com/CJ-Chen/TBtools/releases) (**Figure 1B**). The chromosomal locations were determined by aligning them to the wheat genome database (Ensembl Plants http://plants.ensembl.org/index.html). The results indicated that the *TaHSF* genes were scattered on all 21 chromosomes with the majority of them located at the terminals. These *TaHSFs* were not distributed evenly. While eight *TaHSFs* were located on chromosome 5A, only one was found on each of the chromosomes 1 B, 1 D, 6 A, 6 B, and 6 D. We further analyzed gene duplication events of *TaHSFs* which revealed that genes belonging to *TaHSFA, TaHSFB*, *and TaHSFC* subfamilies might have undergone varying degrees of duplication. Although *TaHSFA* is the largest subfamily among the three *TaHSF* subfamilies, only three gene duplication events were observed. *TaHSFB* and *TaHSFC* subfamilies had witnessed four and seven gene duplication events, respectively. *TaHSFB-5A*, *5B*, and *5D* of the *TaHSFB* subfamily, as well as *TaHSFC1-4*, *C1-5*, and *C1-6* were individually derived from one common ancestor gene (**Figure 1B**).

In wheat, the *TaHSFB4* subfamily is comprised of six members scattered on chromosomes 2 and 5 of subgenomes A, B, and D with one copy on each chromosome. Gene duplication event analysis indicated the duplication events within *TaHsfB4-2* (*TaHsfB4-2A* and *TaHsfB4-2B*) and *TaHsfB4-5* (*TaHsfB4-5A*, *TaHsfB4-5B*, and *TaHsfB4-5D*) genes, respectively (**Figure 1B**). To further explore the evolutionary relationship of TaHSFB4-2B, a phylogenetic tree was constructed using the amino acid sequences of *HSFB4s* from 8 different plant species, including *Dicotyledons*: Arabidopsis (*Arabidopsis thaliana*), soybean (*Glycine max*), tomato (*Solanum lycopersicum*), potato (*Solanum tuberosum*), and rape (*Brassica napus*) and *Monocotyledons*: wheat (*Triticum aestivum*), rice (*Oryza sativa*), and corn (*Zea mayz*) (Ensembl plants database, http://plants.ensembl.org/index.html). For the construction of a phylogenetic tree, the amino acid sequences were aligned using the multiple sequence alignment tool with MEGA-X software (**Figure 1C**). The results showed that TaHSFB4s were clustered in the monocotyledon group, while other HSFB4s were aggregated in the dicotyledon group, suggesting that the evolution of HSFB4s in monocotyledons and dicotyledons was discrepant. These findings were consistent with the results depicted in **Figure 1A**.

### Analysis of abiotic stress-responsive *cis*-elements in *AtHSFs* and *TaHSFs* promoters

**Figure 2:**
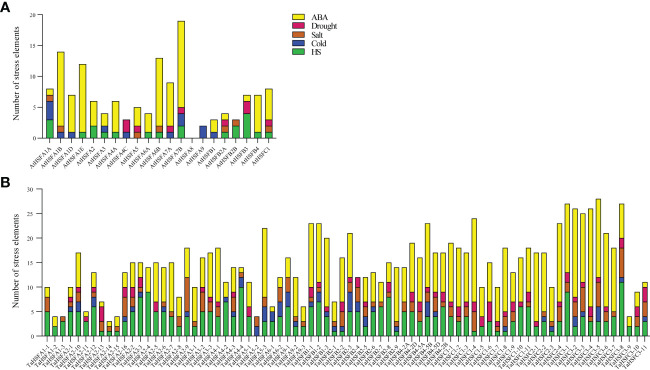
Abiotic stress *cis*-regulating element counts included in promoters of *AtHSFs* and *TaHSFs*. **(A)** The abiotic stress *cis*-element counts included in *AtHSFs* promoters. **(B)** The abiotic stress *cis*-element counts included in *TaHSFs* promoters.

To further study the function of HSFs in Arabidopsis and wheat, the 2 kb region upstream of the start codon of all *AtHSFs* and *TaHSFs* genes was analyzed using the PlantCARE database (http://bioinformatics.psb.ugent.be/webtools/plantcare/html/). The following *cis*-elements responsive to abiotic stresses were chosen; ABA-responsive elements, drought-responsive elements, salt-responsive elements, cold-responsive elements, and heat shock elements (**Figure 2**). In arabidopsis, most of the *AtHSFs* promoters contained 1-4 abiotic stress-responsive elements, except *AtHSFA8*. Of these, *AtHSFA1A, AtHSFA7B, AtHSFB2A*, *and AtHSFC1* contained most of the selected abiotic stress response elements, while *AtHSFA1B, AtHSFA1E, AtHSFA6B*, and *AtHSFA7b* contained the maximum element counts (≥10). Among these abiotic stress response elements, the ABA-responsive element was most abundantly found in many *AtHSF* promoters (**Figure 2A**).

In wheat, all *TaHSF* promoters contained two to five2 abiotic stress response elements. ABA-responsive elements, heat shock elements, and salt-responsive elements were presented in most of the *TaHSF* promoters. Of these, ABA-responsive elements and heat shock elements ranked the top two in terms of quantity of the five response elements (**Figure 2B**). Considering the importance of ABA as one of the major stress-responsive hormones (Mantyla et al., 1995), we speculated that both *AtHSFs* and *TaHSFs* might respond to various abiotic stresses through the pathways mediated by ABA-stress responsive elements along with other elements in plants.

### Expression profiles of *AtHSF* and *TaHSF* genes

**Figure 3:**
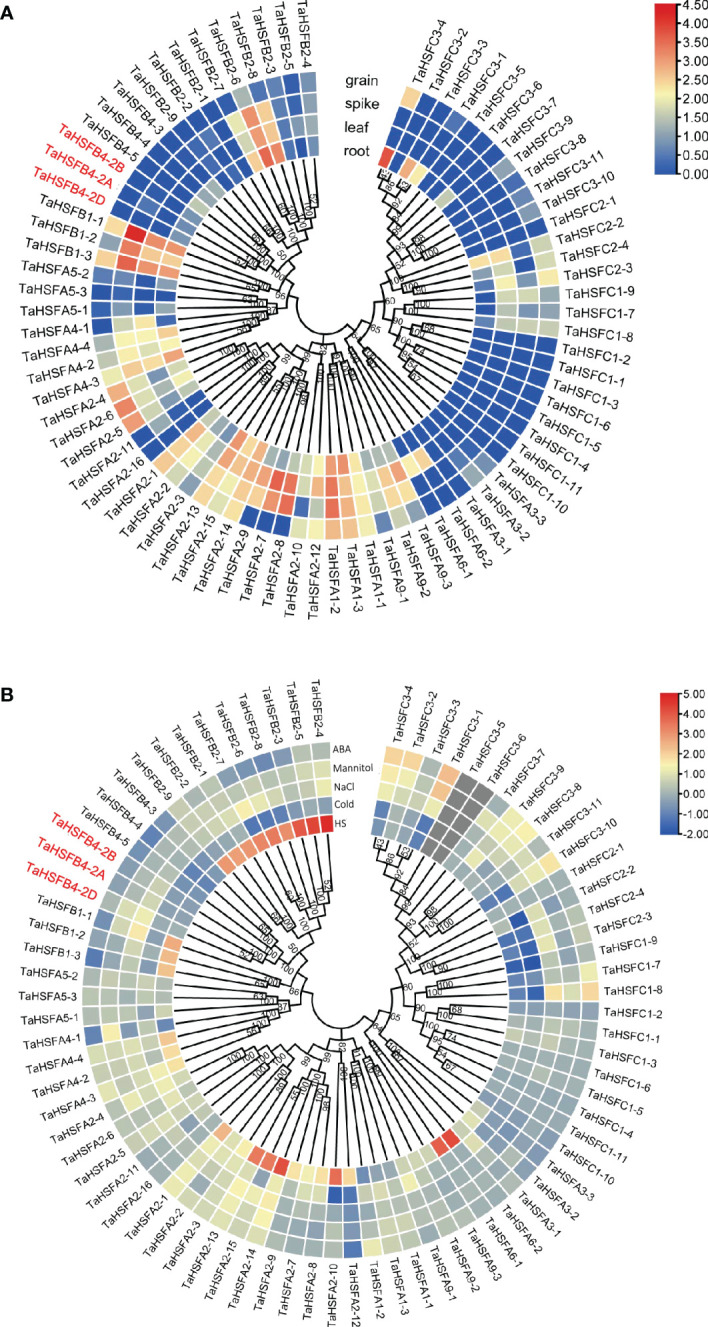
Expression analysis of *TaHSFs* genes in different tissues and stress treatments. **(A)** Expression analysis of *TaHSFs* genes in different tissues based on public data. Bar scale: log_2_TPM. **(B)** Expression analysis of *TaHSFs* genes under different abiotic stress treatments, including heat stress (HS), cold stress (cold), salinity induced stress (NaCl), mannitol-induced dehydration stimulating drought stress (mannitol), and ABA stress (Mittal et al.). Bar scale: log_2_FC, FC: fold change compared with the mock group.

To understand the role of HSFs in plant growth, development, and stress tolerance, we obtained their expression patterns in different tissues and response to abiotic stresses from the arabidopsis and wheat transcriptome data (**Figures S1**, **3**). The arabidopsis expression patterns in response to abiotic stresses and wheat expression patterns were based on the public data (**Figures S1**, **3A**) (http://plants.ensembl.org/index.html). The expression profiles of *TaHSFs* in response to different abiotic stress treatments were investigated in our laboratory (**Figure 3B**). The expression levels of *AtHSFs* varied greatly, both in case of one gene among different tissues and different genes in the same tissue in arabidopsis, with exceptions that *AtHSF6A*, *AtHSF6B*, and *AtHSFB3* showed almost no expression in all tested tissues. While *AtHSFA9* was highly expressed only in dry and stage 9 seeds*, AtHSFA7B* was transcribed moderately in dry seeds, imbibed seeds, and roots, indicating that *AtHSFs* may perform distinct functions in plant growth and development (**Figure S1A**). We next analyzed the expression of *AtHSFs genes* in response to heat shock, cold, NaCl, and mannitol-induced stress. Since ABA is a stress-responsive hormone, the expression analysis of *AtHSFs* in response to exogenous ABA was also investigated in our study. The expression of most *AtHSFs* was unaltered by cold, NaCl, or mannitol-induced stress, while transcription of the remaining *At*HSFs was mildly suppressed. These observations suggested that only a few *AtHSFs* are involved in the pathways imparting tolerance to cold, salinity, and mannitol-induced stress in arabidopsis. After heat shock treatment, the expression of *AtHSFA2*, *AtHSFA7A*, and *AtHSFA7B*, especially *AtHSFA7B*, was significantly increased. In contrast, heat shock treatment repressed the transcription of *AtHSFC1* and *AtHSFA1A.* Further, ABA strongly induced the expression of *AtHSFA6A* and *AtHSFA6B* but reduced the expression of *AtHSFA3* and *AtHSFA7B* (**Figure S1B**). These results indicated that since the expression of *AtHSFs* varies in response to different abiotic stresses, their function might also have diverged.

Unlike *AtHSFs*, the tissue-specific expression analysis of 34 *TaHSFAs* indicated that the genes expressed at different levels in all investigated wheat tissues. As an exception, *TaHSFA3-1, -2, -3, TaHSFA2, -16*, and *TaHSFA5-3* expressed at very low or almost non-detectable levels. Most *TaHSFCs* genes are expressed at low levels in these tissues. Precisely, *TaHSFBs, TaHSFB1-1, -2, -3*, and *TaHSFB2-6, -7, -8* were robustly expressed in root, leaf, and spike. *TaHSFB1-1 and -3* were also found to be expressed in grain. Other *TaHSFBs* did not show expression in the examined tissues (**Figure 3A**). Only heat shock-induced expression of some *TaHSFA* and *TaHSFB* genes, especially *TaHSFA2-10, -13, -14, -15*, *TaHSFA6-1, -2*, and *TaHSFB2*, was observed whose transcription levels were upregulated. On the contrary, cold, NaCl, and mannitol-induced stress and exogenous ABA treatment did not show a strong impact on *TaHSFs* expression (**Figure 3B**).

### Amino acid sequence alignment and structural analysis of TaHSFB4-2B

**Figure 4:**
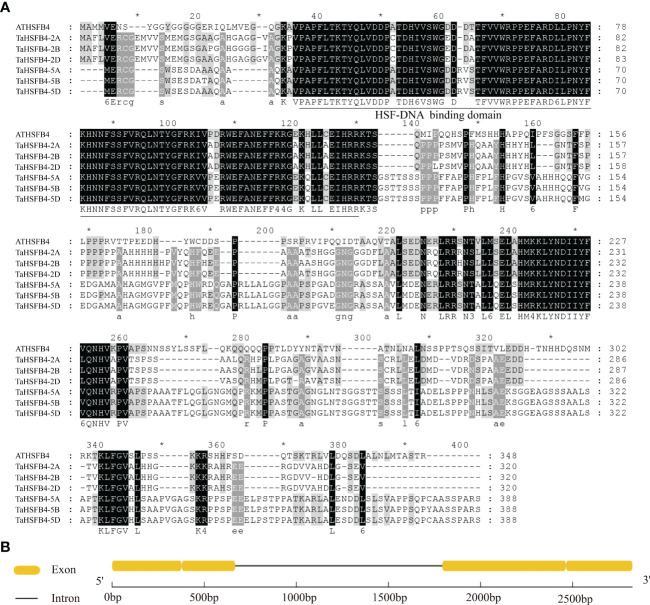
Amino acid sequence alignment of HSFB4s subfamily members. **(A)** Multiple sequence alignment of predicted amino acid sequence of TaHSFBs and AtHSFB4. Grey represents different degrees of conservation among sequences, black indicates identical residues, white indicates conservative changes. The conserved HSF-DNA binding domain was underlined. **(B)** *TaHSFB4-2B* gene sequence structure.

Among the six TaHSFB4s in wheat, including TaHSFB4-2A, TaHSFB4-2B, TaHSFB4-2D, TaHSFB4-5A, TaHSFB4-5B, and TaHSFB4-5D, TaHSFB4-2B exhibited the highest sequence homology with AtHSFB4. Therefore, TaHSFB4-2B was selected for further characterization, and the encoding gene sequence was cloned through homology-based cloning (**Figure 4A**). In addition, > 90% sequence homology was detected among the wheat homologs of TaHSFB4 on the ABD subgenomes, namely TaHSFB4-2A, TaHSFB4-2B, and TaHSFB4-2D. Moreover, the homology between TaHSFB4-2B and other TaHSFB4s, including TaHSFB4-5A, TaHSFB4-5B, and TaHSFB4-5D, was around 80%, indicating that the TaHSFB4 subfamily was highly conserved in wheat.

The structure of TaHSFB4-2B is comprised of two exons and one intron (**Figure 4B**). Coding sequence (CDS) of *TaHSFB4-2B* contains 963 base pairs (bp), which encodes 320 amino acids. The protein structure prediction indicated that the putative protein contains an HSF-DNA binding domain at the N-terminal (**Figure 4A**).

### Subcellular localization of TaHSFB4-2B in *T. aestivum* and *A. thaliana* protoplast and its tissue-specific expression analysis in wheat

**Figure 5:**
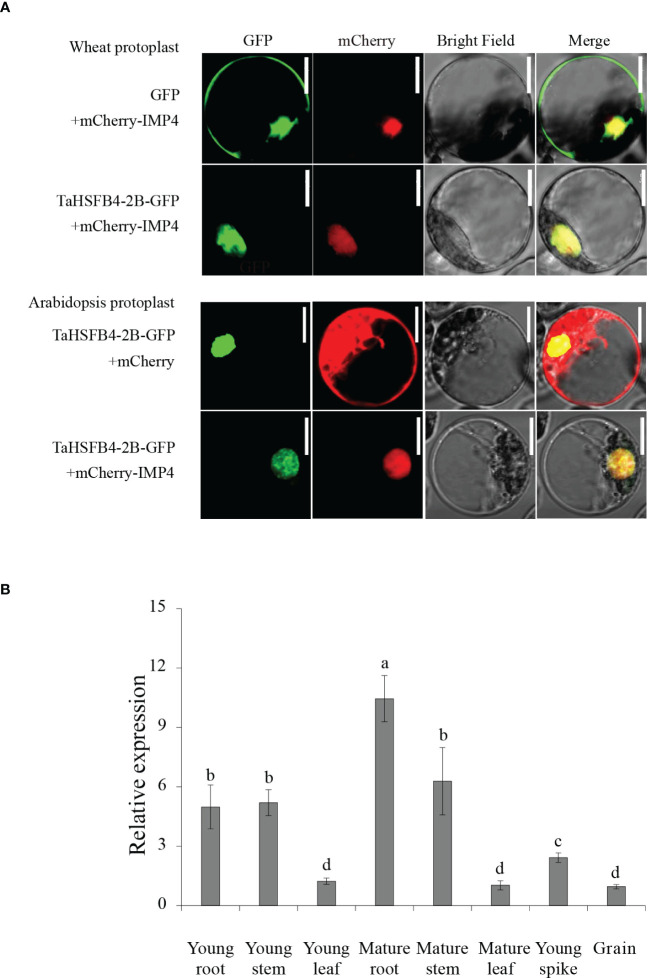
TaHSFB4-2B subcellular localization in *Triticum aestivum* and *Arabidopsis thaliana* protoplast and *TaHSFB4-2B* tissue specific expression profiles. **(A)** TaHSFB4-2B Subcellular localization in *Triticum aestivum* and *Arabidopsis thaliana* protoplast. mCherry-IMP4 was used as nuclear marker. Row 1: Free GFP and mCherry-IMP4 were transiently expressed in *Triticum aestivum* protoplast; Row 2: TaHSFB4-2B-GFP and mCherry-IMP4 were transiently expressed in *Triticum aestivum* protoplast; Row 3: TaHSFB4-2B-GFP and free mCherry were transiently expressed in *Arabidopsis thaliana* protoplast; Row 4: TaHSFB4-2B-GFP and mCherry-IMP4 were transiently expressed in *Arabidopsis thaliana* protoplast. Green channel: GFP fluorescence signals; Red channel: mCherry fluorescence signals; Scale bar: 10 μm. **(B)** *TaHSFB4-2B* tissue specific expression profiles in wheat. The young root, mature root, young stem, mature stem, young leaf, mature leaf, young spike and grain of wheat at different growth stages were sampled the transcription levels of *TaHSFB4-2B* were measured by RT-qPCR. The transcription level of *TaHSFB4-2B* was normalized with *TaACTIN*. Values are Mean ± SD, n=3. a, b, c and d indicate the statistical differences between different tissues determined by LSD (*P* < 0.05) of variance (ANOVA) method. Same letters: no significant difference, and different letters: significant difference between the two groups.

Previous studies have shown that TaHSFB4-2B is located in the nucleus (Li-Na et al., 2018). Protein domain prediction also indicated that TaHSFB4-2B is a transcription factor with an HSF-DNA binding domain, that is putatively localized in the nucleus. To investigate the subcellular localization of TaHSFB4-2B, the cassette encoding TaHSFB4-2B-Green Fluorescent protein (GFP) fusion protein driven by the CaMV 35S promoter (*35S*::TaHSFB4-2B-GFP) was transformed into wheat and Arabidopsis protoplast or *Nicotiana benthamiana* leaves, and the fluorescence was observed using the confocal microscope. Results indicated that TaHSFB4-2B-GFP was localized in the nucleus and showed co-localization with nuclear marker mChery-IMP4 (**Figures 5A**, **S4**). In addition, TaHSFB4-2B nuclear localization was also proved by visualization of TaHSFB4-2B-GFP in transgenic Arabidopsis seedlings (**Figure S3**).

Gene function is largely affected by its specific location of expression in the whole plant. Depending on the tissue-specific expression analysis in wheat, *TaHSFB4-2B* was found to be moderately expressed in wheat roots but barely expressed in the leaf, spike, and grain (**Figure 3A**). To further validate and determine the tissue-specific expression of *TaHSFB4-2B*, samples of the young root, mature root, young stem, mature stem, young leaf, mature leaf, young spike, and young seed were collected, and the transcription levels of *TaHSFB4-2B* were measured by real-time quantitative reverse transcription PCR (RT-qPCR). *TaHSFB4-2B* transcript was expressed in all the tissues with various expression levels (**Figure 5B**). The highest transcription level was in the mature root, followed by the mature stem, young stem, and young root, and the lowest was found in the mature leaf. This was consistent with the results of the tissue-specific analysis (**Figure 3A**).

### Expression analysis of *TaHSFB4-2B* under salinity and mannitol-induced stresses in wheat roots and young leaf

**Figure 6:**
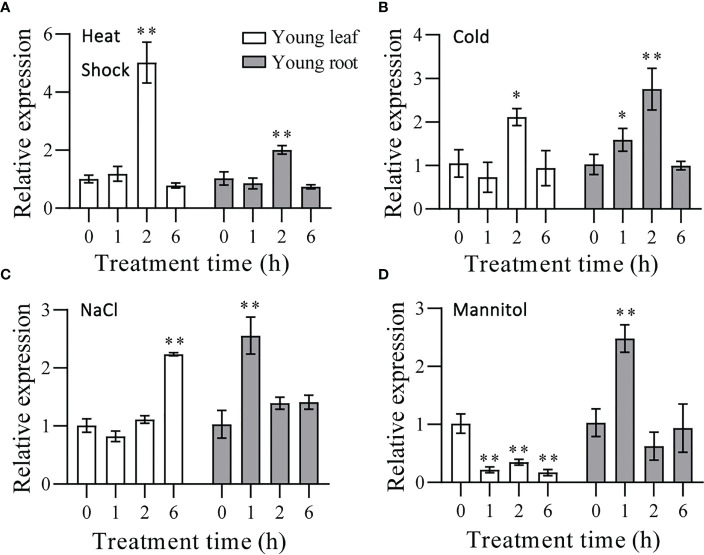
Expression levels of *TaHSFB4-2B* in wheat seedlings young leaf and young root of wheat under abiotic stress. **(A)** Wheat seedlings growing for 14 d after germination were treated under high temperature (37°C). **(B)** Cold stress (4°C). **(C)** NaCl induced stress (200 mM NaCl). **(D)** mannitol-induced dehydration stimulating drought stress (300 mM mannitol). Young leaf and young root were sampled after 0, 1, 2, and 6h of each treatment and the transcription levels of *TaHSFB4-2B* were quantified by RT-qPCR. The transcription level of *TaHSFB4-2B* was normalized with *TaACTIN*. Values are Mean ± SD, n=3. *P < 0.05 and **P < 0.01 (Student’s t-test).

Wheat seedlings exposed to NaCl and mannitol showed increased transcription of *TaHSFB4-2B*, while heat shock, cold, and ABA treatment exerted little effect on *TaHSFB4-2B* expression (**Figure 3B**). To verify the results, we performed RT-qPCR analysis of *TaHSFB4-2B* in seedlings treated with heat shock (37°C), cold (4°C), salinity stress (200 mM NaCl), and mannitol treatment (300 mM mannitol). Heat shock and cold treatment induced the transcription of *TaHSFB4-2B* in both the young leaf and root (**Figures 6A, B**). Though the expression of *TaHSFB4-2B* was also induced by salinity stress in both leaves and roots, the time course reaching the peak level was discrepant (**Figure 6C**). After mannitol-induced drought treatment, the expression of *TaHSFB4-2B* was down-regulated in young leaf, but up-regulated in the young root (**Figure 6D**). In brief, our results indicated that Heat Shock, Cold, NaCl, and mannitol treatments induced the expression of *TaHSFB4-2B* in young root and the young leaf of wheat, while *TaHSFB4-2B* transcription level was repressed in young leaf with mannitol treatment.

### Overexpressing of TaHSFB4-2B negatively regulates the tolerance of Arabidopsis seedlings to NaCl and mannitol-induced stresses

**Figure 7:**
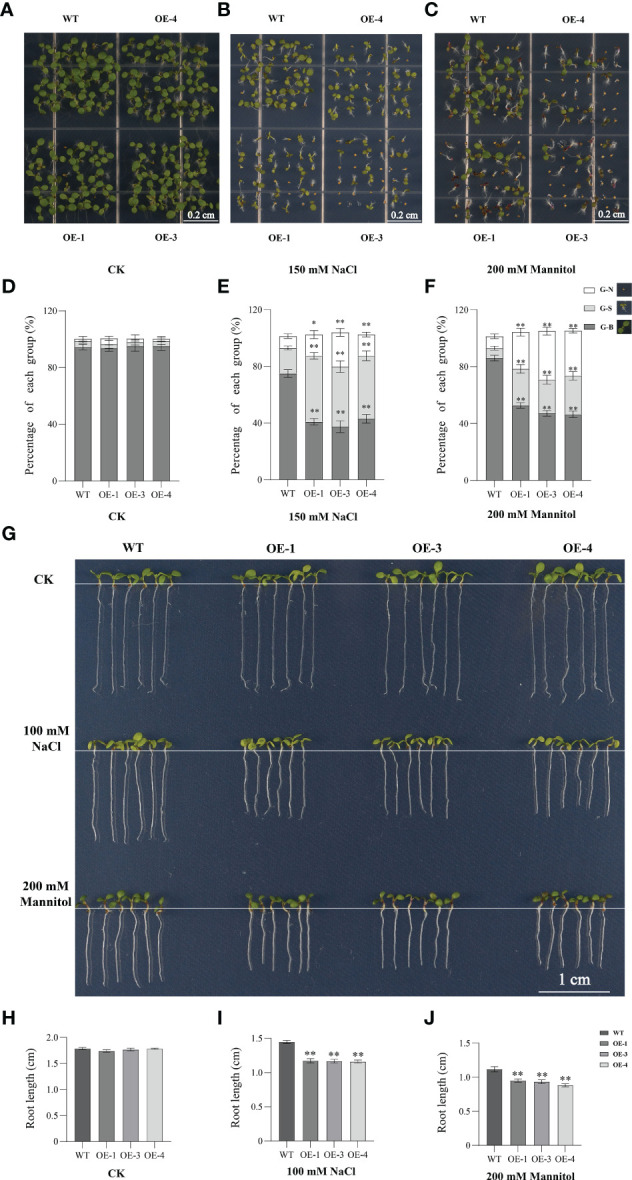
Overexpression of *TaHSFB4-2B* negatively regulates NaCl, and mannitol induced stress tolerance of arabidopsis. **(A–C)** Seeds of WT and *TaHSFB4-2B* overexpression lines of arabidopsis (OE-1, OE-3 and OE-4) were sterilized and plated on 1/2 Murashige and Skoog medium (1/2 MS) plates or with 150 mM NaCl or with 200 mM mannitol and growing for 10 days. **(D, E)** The seed germination rates and growth under corresponding treatment were quantified. **(G–J)** Root phenotypes of wild type and OE-1, OE-3 and OE-4 lines were shown and root length was measured and quantified. G-N、G-S and G-B represent ungerminated seeds, inhibited-growing seedlings and normal-growing seedlings respectively. Values are Mean ± SD, n=3. *P < 0.05 and **P < 0.01 (Student’s t-test).

To elucidate the biological function of TaHSFB4-2B in response to abiotic stresses, *35S*::*TaHSFB4-2B-GFP* cascade was constructed and transformed into arabidopsis using *Agrobacterium*. Transgenic lines (*TaHSFB4-2B*-OEs) were genotyped by PCR amplification (**Figure S2A**) and the expression level of TaHSFB4-2B was quantified by RT-qPCR (**Figure S2B**). Subsequently, low (OE-1), medium (OE-3), and high (OE-5) expression lines were selected for further research. Visualization of GFP fusion protein signals under a confocal microscope verified the expression and nucleus localization of *TaHSFB4-2B* (**Figure S3**). The phenotyping results indicated that overexpression of *TaHSFB4-2B* had no discernible effect on plant growth and development under normal conditions (**Figures 7A, G**).

To explore the function of *TaHSFB4-2B* under salinity and mannitol-induced drought stresses, WT and *TaHSFB4-2B*-OE seedlings were continuously grown on 1/2 MS plates with or without NaCl (150 mM) or mannitol (200 mM) for 10 days. Then the seed germination rates were calculated and root length was measured (**Figure 7**). Both NaCl and mannitol treatment decreased the seed germination and root length in WT as well as *TaHSFB4-2B*-OE lines. Moreover, *TaHSFB4-2B*-OE lines were more sensitive to NaCl and mannitol exposure than WT (**Figure 7**). Compared to the WT, NaCl treatment reduced seed germination in *TaHSFB4-2B*-OE lines by 18.1 to 42.4% (**Figures 7A, B, D, E**); while the root was decreased by 16.2 to 19.0% in the transgenic lines (**Figures 7G–I**). Similar phenotypes related to seed germination and root length were observed with mannitol treatment (**Figures 7A, C, F–H, J**). These results indicated that both NaCl and mannitol treatment negatively affect seed germination and root length in arabidopsis, and *TaHSFB4-2B*-OE lines were more sensitive to both NaCl and mannitol-induced stress than WT.

### Expression analysis of abiotic stress-associated genes in *TaHSFB4-2B*-OE Arabidopsis lines

**Figure 8:**
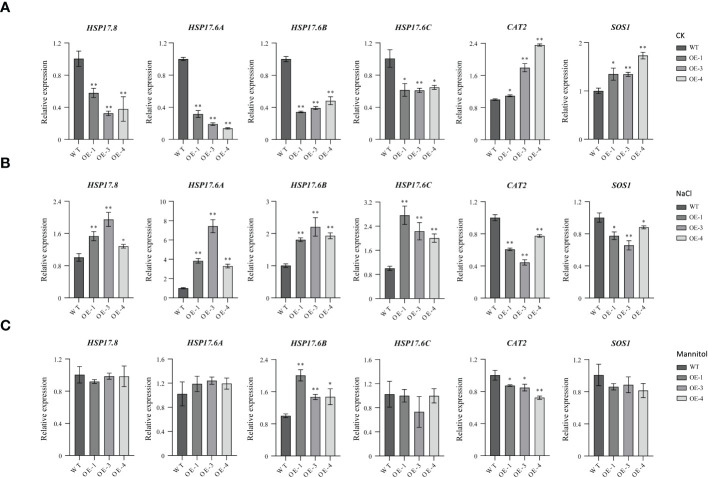
Expression analyses of abiotic stress related genes in WT and *TaHSFB4-2B*-OE lines under different abiotic stress. Seeds of WT and *TaHSFB4-2B* over expression lines of arabidopsis (OE-1, OE-3 and OE-4) were growing and treated and use method described in **Figure 7**. **(A)** Transcription levels of *AtHSP17.8*, *AtHSP17.6A*, *AtHSP17.6B*, *AtHSP17.6C*, *CAT2* and *SOS1* growing under normal condition (CK), **(B)** NaCl treatment (100 mM NaCl), **(C)** mannitol treatment (200 mM mannitol) were quantified by RT-qPCR. The transcription level of *TaHSFB4-2B* was normalized with *AtACTIN2*. Values are Mean ± SD, n=3. *P < 0.05 and **P < 0.01 (Student’s t-test).

Since overexpression of *TaHSFB4-2B* repressed the tolerance of arabidopsis to mannitol and NaCl-induced stresses, RT-qPCR analysis was performed to quantify the transcription levels of abiotic stress-induced genes, including *AtHSP17.8*, *AtHSP17.6A*, *AtHSP17.6B*, *AtHSP17.6C*, *SOS1*, and *CAT2.* Since *TaHSFB4-2B* is a heat shock factor, expression analysis of small heat shock proteins, including *AtHSP17.8*, *AtHSP17.6A*, *AtHSP17.6B*, and *AtHSP17.6C*, was also performed. The results showed that the transcription of four small *HSPs* was down-regulated in *TaHSFB4-2B*-OE lines in mock groups (CK), but up-regulated with NaCl treatment (**Figures 8A, B**). With mannitol treatment, only the expression of *AtHSP17.6B* was increased in *TaHSFB4-2B*-OE lines compared to WT, while the expression levels of *AtHSP17.8*, *AtHSP17.6A*, and *AtHSP17.6C* were unaffected (**Figure 8C**). Overexpression of *TaHSFB4-2B* induced the expression of *CAT2* and *SOS1* genes in arabidopsis. In contrast, expression levels of *CAT2* and *SOS1* were reduced when exposed to NaCl or mannitol, except *SOS1* expression with mannitol treatment (**Figure 8**).

## Discussion

Heat shock transcription factors (*HSFAs, HSFBs*, and *HSFCs*) are multifunctional genes engaged in plant growth and development as well as abiotic stress responses (Kotak et al., 2007; Lin et al., 2011; Scharf et al., 2012). The numbers of *HSF* gene family members showed a large variation with 78, 31, 21, and 26 in wheat, maize, arabidopsis and tomato, respectively. The phylogenetic relationship of corresponding homologous proteins within each subfamily was closer within monocotyledon or dicotyledon plants than between the both (**Figure 1A**). In different plants, *HSF* genes experienced extensive duplication and sequence variation during evolution, indicating that *HSFs* perform conserved and diverged functions in plants (**Figure 1B**). Analysis of abiotic stress-associated *cis*-elements in *AtHSF* and *TaHSF* promoters revealed the presence of one or more *cis*-elements responsive to ABA, NaCl, mannitol, cold, and/or heat shock. The ABA-responsive elements were present in most of the *AtHSF* and *TaHSF* promoters. Interestingly, *TaHSF* promoters contain heat shock and salt response elements at a higher proportion than those of *AtHSFs* (**Figure 2**). We speculate that regulation of *HSFs* in different plants shows certain conservation, and at the same time, variations have evolved. Therefore, the expression of HSF genes varies in different tissues or at developmental stages in both arabidopsis and wheat. The majority of both arabidopsis and wheat *HSFA* genes showed robust transcription in tested tissues at different stages. However, *TaHSFBs* and *TaHSFCs* exhibited very low expression in most of the conditions (**Figures S1A**, **3A**). We infer that *HSFAs* function more extensively and actively compared to *HSFBs* and *HSFCs* in plant development and growth.

Phylogenetic analysis classified *HSFB4s from* monocotyledons and dicotyledons into different groups (**Figure 1C**). Sequence similarity analysis indicated that *TaHSFB4s* did not share significant similarity with *ATHSFB4* (**Figure 4A**). We speculate that the biological function of these *TaHSFB4s* are conserved in wheat, but may differ from dicotyledons. Previous studies and our expression pattern analysis showed that *AtHSFB4* and *TaHSFB4s* expressed extensively in different tissues (**Figures S1A**, **3A**) (Xue et al., 2014). In this study, *TaHSFB4-2B* was cloned from the wheat variety Chinese Spring using a homology-based cloning method. Tissue-specific expression analysis using RT-qPCR showed that *TaHSFB4-2B* was expressed in the root, stem, leaf, young spike, and the young seed of wheat, and the transcription level varied in different tissues (**Figure 5B**). These observations highlighted that *TaHSFB4-2B* may function in the whole plant and at different stages throughout the wheat life cycle. TaHSFB4-2B was found to be localized in the nucleus (**Figure 5A**) and harbored an HSF-DNA binding domain (**Figure 4A**). However, TaHSFB4-2B lacks a transcriptional activation domain, therefore, we suspect that *TaHSFB4-2B* probably interacts with other proteins to regulate the transcription of downstream genes.

A big number of *AtHSFs* and *TaHSFs* were greatly induced by heat shock treatment. Heat shock, cold, NaCl, mannitol, and ABA treatment moderately induced the expression of *AtHSFBs* in arabidopsis, including *AtHSFB4* (**Figures S1B**, **3B**). Recent studies have also indicated that *TaHSFs*, including *TaHSFB4*, play a key role in enhancing tolerance to various abiotic stresses (Duan et al., 2019; Duan et al., 2019). Our results showed the upregulated transcription of *TaHSFB4-2B* under the above-mentioned abiotic stress treatments (except in leaf under mock drought stress), substantiating that *TaHSFB4-2B* is involved in abiotic stress responses (**Figure 6**).

Earlier, significant differences have been observed in the functions of *HSFs* in different plants. Overexpression of *OsHSFB2b* in transgenic arabidopsis reduces salt and drought tolerance, while overexpression of *CarHSFB2* significantly improves heat and drought tolerance (Ma et al., 2015). Ectopic expression of wheat *TaHSF3* in arabidopsis. improved heat and cold tolerance in transgenic plants (Zhang et al., 2012). In arabidopsis, *AtHsfB4* was been reported in regulating root development, and had few effects in stress responses (Begum et al., 2013). In our research, we also found the *AtHsfB4* expression was barely induced by stress treatment in arabidopsis (**Figure S1**). On the other hand, the wheat *TaHsB4* expression was elevated by drought and salinity (**Figure 3**). Consist with this, our research indicated that the ectopic over expression of wheat *TaHsfB4* showed no difference in root at normal condition (**Figure 7**). Then we tried to check the resistance of *TaHSFB4-2B-GFP* overexpression line in arabidopsis under mannitol and salinity condition. The seed germination rate of *TaHSFB4-2B*-OE lines was significantly reduced and the root length was shortened under NaCl and mannitol-induced stress (**Figure 7**). Taken together, overexpression of *TaHSFB4-2B* negatively regulates the salinity and mannitol-induced drought tolerance in arabidopsis.

The decreased transcription levels of *CAT2* and *SOS1* in *TaHSFB4-2B-OE* lines under salinity and mannitol-induced drought stress were consistent with the phenotypes (**Figures 8B, C**). *HSPs* are by far the most complex heat shock proteins in plants. Due to their abundance and diversity, *HSPs* play an important role in plant stress tolerance. Plant *HSFs* regulate the expression of *HSPs* in response to abiotic stress (Nover et al., 1996). Overexpression of *Agrostis stolonifera HSP17* reduced NaCl and mannitol-induced dehydration stimulating drought tolerance in arabidopsis, suggesting that excessive *AsHSP17* itself is a repressor of salt and drought stress response (Sun et al., 2016). Overexpression of *AtHSP17.8* in arabidopsis and *Lactuca sativa* resulted in hypersensitivity to ABA and enhanced tolerance to mannitol and NaCl-induced stresses (Kim et al., 2013). In the current study, we found that in *TaHSFB4-2B-*OE lines, the transcription levels of *AtHSP17.8, AtHSP17.6A*, *AtHSP17.6B*, and *AtHSP17.6C* were significantly up-regulated under NaCl-induced salinity stress (**Figure 8B**). While, under mannitol-induced stress, only the expression of *AtHSP17.6B* was increased, transcription levels of *AtHSP17.8*, *AtHSP17.6A*, and *AtHSP17.6C* were unaffected (**Figure 8C**). These observations manifested that *AtHSP17.8, AtHSP17.6A*, and *AtHSP17.6C* participate in plant response to salinity stress, and *AtHSP17.6B* is involved in tolerance to both salinity and mannitol-induced stresses.

Surprisingly, the expression of *AtHSP17.8, AtHSP17.6A*, *AtHSP17.6C, CAT2*, and *SOS1* in our study showed contrasting profiles between normal and stress conditions (Except for the expression of *AtHSP17.8*, *AtHSP17.6A*, and *AtHSP17.6C* under drought stress, which showed similar transcription level in both normal and drought stress conditions) (**Figure 8**). Considering the observation that *TaHSFB4-2B* is a transcription factor without the transcriptional activation activity, it should interact with other proteins to regulate the expression of downstream genes. It is speculated that *TaHSFB4-2B* combines different regulatory factors under different environmental stimuli to affect gene expression. It is interesting to further screen these regulatory factors and study the mechanism underlying gene expression regulation in the future.

## Conclusion

The evolutionary analysis revealed that it is clustered in a group with monocotyledons. The results of the laser scanning confocal microscope showed that TaHSFB4-2B was located in the nucleus. Tissue-specific expression analysis indicated that the transcription level of *TaHSFB4-2B* was higher in roots and stems and relatively lower in leaves. Overall, our study demonstrated that *the TaHSFB4-2B* gene responds to high temperature, cold, salinity, and mannitol-induced drought stress. Overexpression of *TaHSFB4-2B* reduced the salinity and mannitol-induced drought stress tolerance in transgenic arabidopsis and affected the expression of abiotic stress-related genes. We propose that *TaHSFB4-2B* functions as a negative factor to abiotic stress tolerance in plants, especially to NaCl and mannitol-induced stresses. The biological function of *TaHSFB4-2B* in abiotic stress response and the underlying mechanism deserves detailed study.

## Data availability statement

The data presented in the study are deposited in the NCBI repository, accession numbers SAMN31092595 - SAMN31092606.

## Author contributions

JG and XZ conceptualized the study. XZ, JG and ZZ designed the project and supervised the study. LY and YZ performed most of the experiment and material preparation. JyY and CW performed part of the experiment and offered help with manuscript revision. SL and LJ performed data analyses and interpretation. JhY and LS participated in some experiment. LZ and YS guided part of experiment and data analyses. LY prepared the draft of the manuscript, JG and XZ revised the manuscript and figures. All authors contributed to the article and approved the submitted version.

## Supplementary material

Supplementary Figure 1 Expression analysis of *AtHSFs* genes in different tissues of different developmental stages and stress treatments based on public data. **(A)** Expression analysis of *AtHSF* genes in different tissues at different stages. Bar scale: log_2_TPM. **(B)** Expression analysis of *AtHSF* genes under different abiotic stress treatments, including heat shock stress (HS), cold stress (cold), NaCl induced stress (NaCl), mannitol induced stress (mannitol), and ABA stress (Mittal et al.). Bar scale: log_2_FC, FC: fold change compared with mock group.

Supplementary Figure 2 Genotyping of *TaHSFB4-2B* overexpressing plants. **(A)** PCR genotyping of *TaHSFB4-2B* overexpressing lines with *TaHSFB4-2B* specific primers. **(B)** RT-qPCR analysis of *TaHSFB4-2B* transcription level in wild type and *TaHSFB4-2B* overexpressing lines of Arabidopsis. OE-1, OE-2, OE-3, OE-4, OE-5 indicate different transgenic lines overexpressing *TaHSFB4-2B*.

Supplementary Figure 3 Fluorescence microscopic observation of root of five transgenic Arabidopsis lines. Scale: 20 μm.

Supplementary Figure 4 TaHSFB4-2B subcellular localization in *Nicotiana Benthamiana* leaf. mCherry-IMP4 was used as nuclear marker. TaHSFB4-2B-GFP fusion protein driven by the CaMV35S promoter was transiently expressed in *Nicotiana Benthamiana* leaf. Green channel: GFP fluorescence signals. Red channel: mCherry fluorescence signals. Scale bar: 20 μm.
