# Supplementary figures and images for: Phylogenetic and expression analyses of HSF gene families in wheat (Triticum aestivum L.) and characterization of TaHSFB4-2B under abiotic stress
Source: Front Plant Sci. 2023 Jan 25;13:1047400. doi: 10.3389/fpls.2022.1047400 (PMC9905432; doi:10.3389/fpls.2022.1047400)

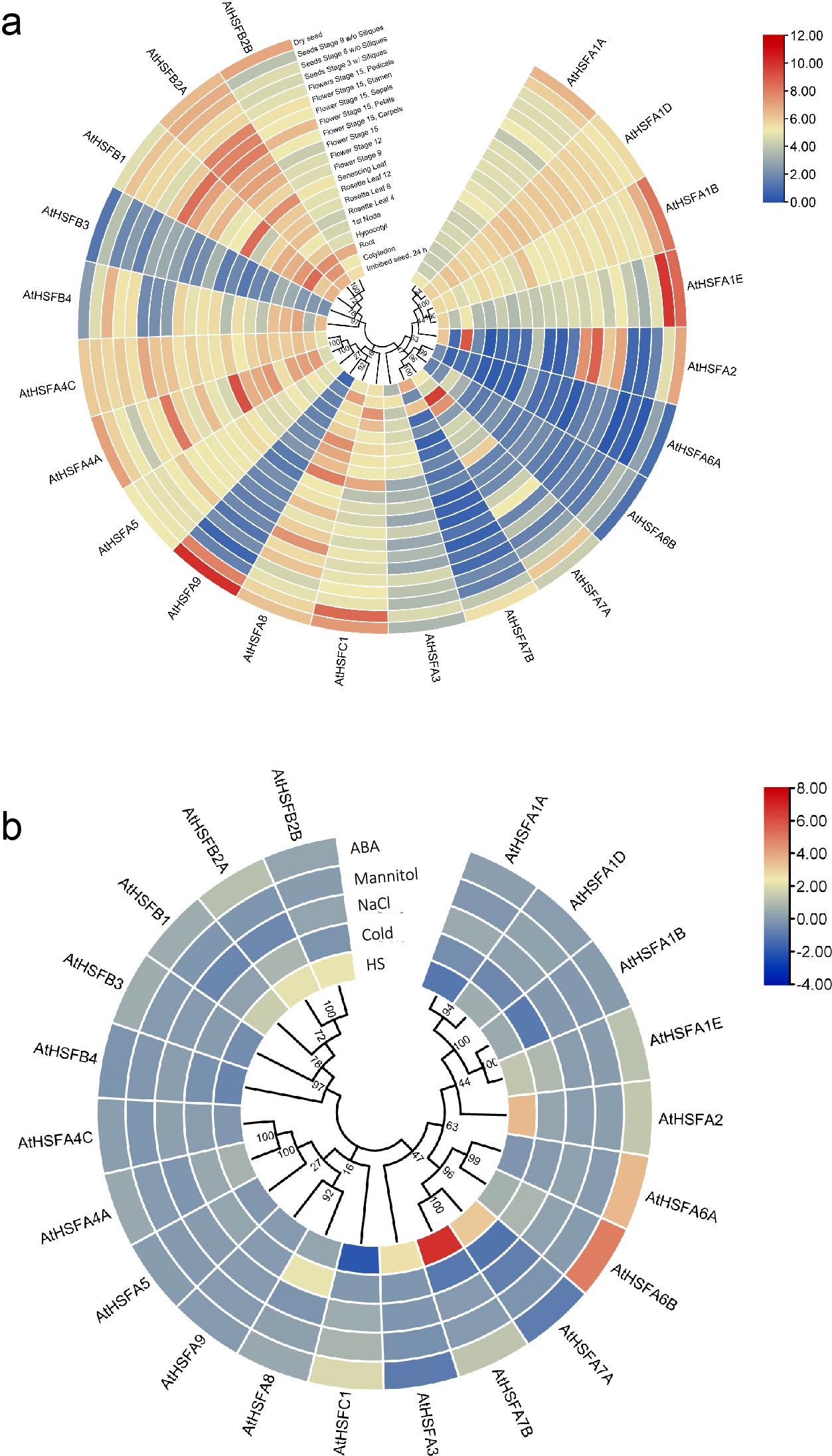

Supplement: Supplementary Figure 1 — Expression analysis of AtHSFs genes in different tissues of different developmental stages and stress treatments based on public data. (A) Expression analysis of AtHSF genes in different tissues at different stages. Bar scale: log2TPM. (B) Expression analysis of AtHSF genes under different abiotic stress treatments, including heat shock stress (HS), cold stress (cold), NaCl induced stress (NaCl), mannitol induced stress (mannitol), and ABA stress (Mittal et al.). Bar scale: log2FC, FC: fold change compared with mock group. [file Image_1.jpeg]

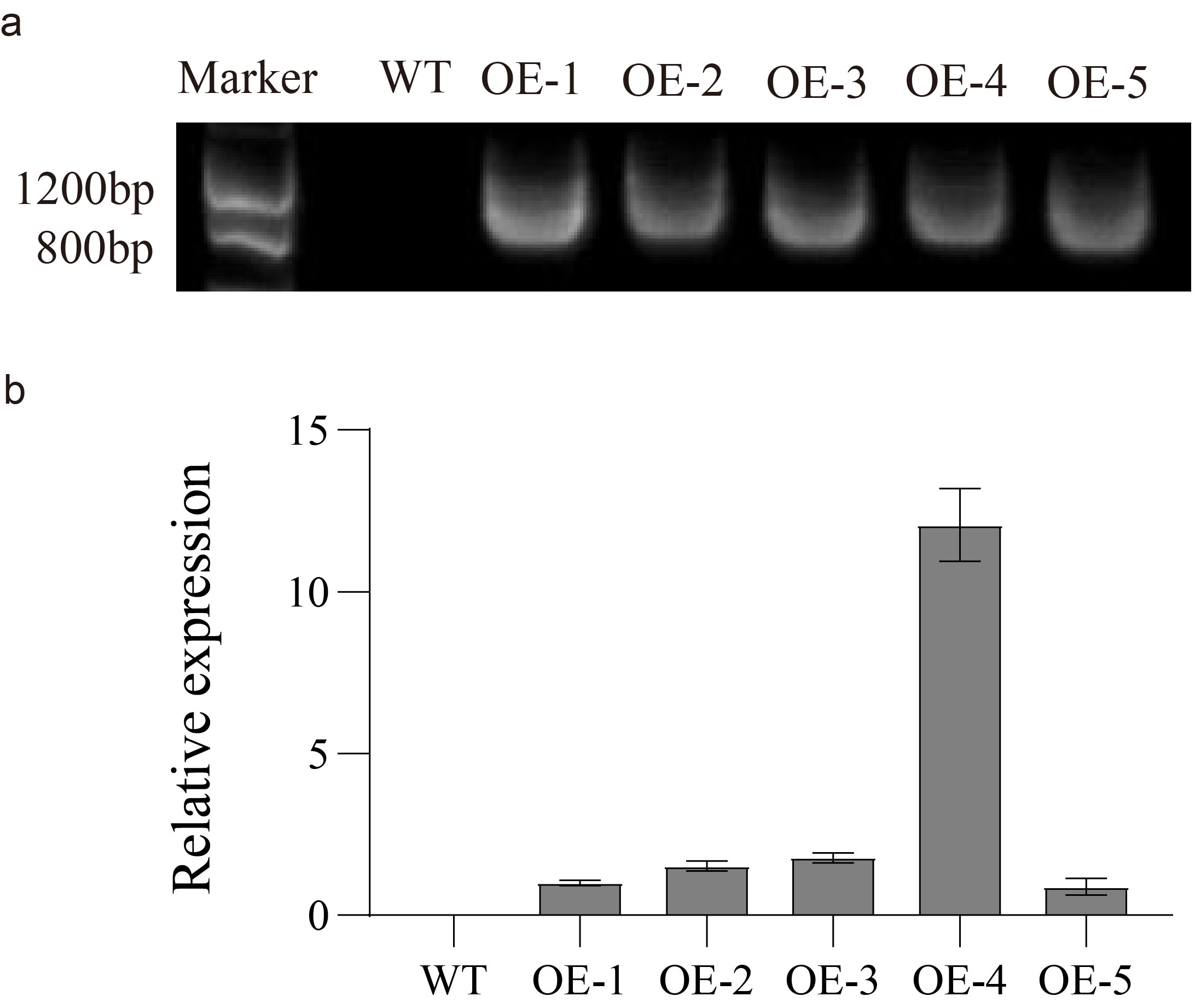

Supplement: Supplementary Figure 2 — Genotyping of TaHSFB4-2B overexpressing plants. (A) PCR genotyping of TaHSFB4-2B overexpressing lines with TaHSFB4-2B specific primers. (B) RT-qPCR analysis of TaHSFB4-2B transcription level in wild type and TaHSFB4-2B overexpressing lines of Arabidopsis. OE-1, OE-2, OE-3, OE-4, OE-5 indicate different transgenic lines overexpressing TaHSFB4-2B. [file Image_2.jpeg]

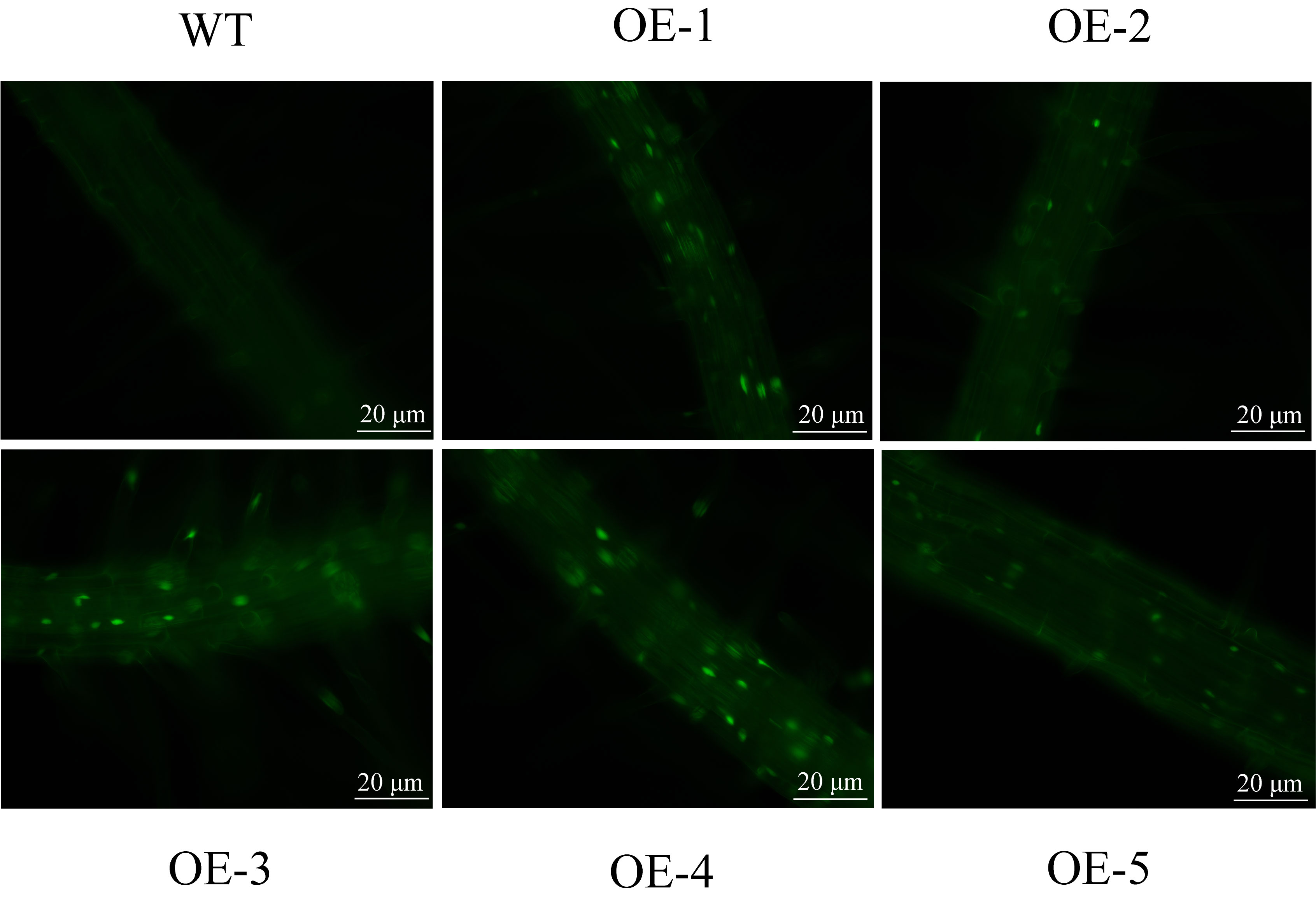

Supplement: Supplementary Figure 3 — Fluorescence microscopic observation of root of five transgenic Arabidopsis lines. Scale: 20 μm. [file Image_3.jpeg]

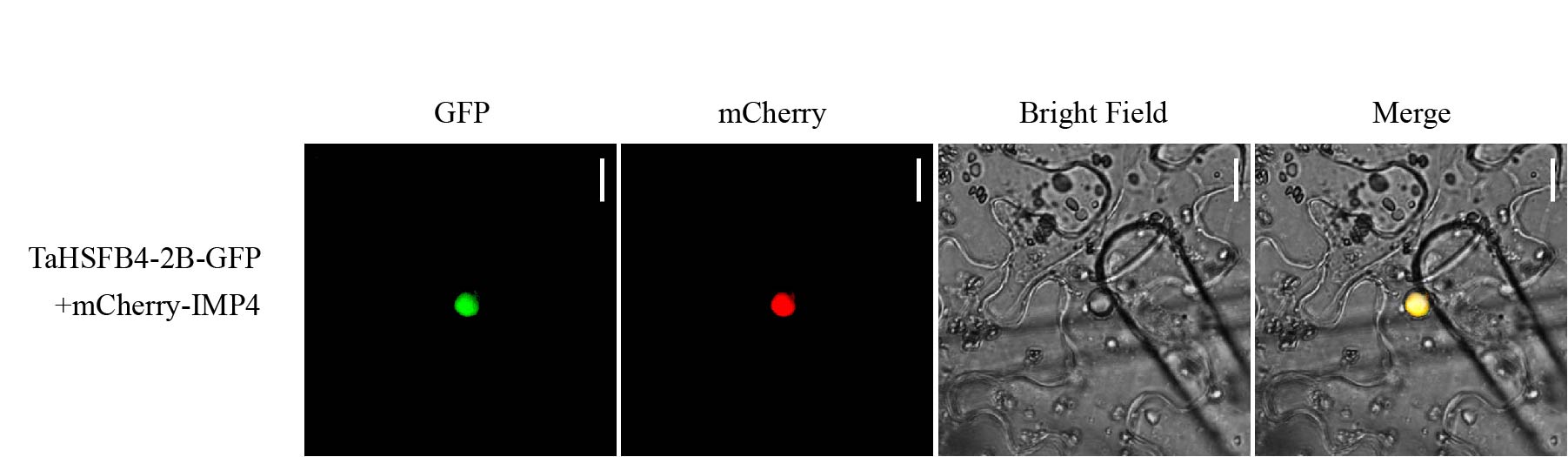

Supplement: Supplementary Figure 4 — TaHSFB4-2B subcellular localization in Nicotiana Benthamiana leaf. mCherry-IMP4 was used as nuclear marker. TaHSFB4-2B-GFP fusion protein driven by the CaMV35S promoter was transiently expressed in Nicotiana Benthamiana leaf. Green channel: GFP fluorescence signals. Red channel: mCherry fluorescence signals. Scale bar: 20 μm. [file Image_4.jpeg]
